# Low-chloride- versus high-chloride-containing hypertonic solution for the treatment of subarachnoid hemorrhage–related complications: The ACETatE (A low ChloriE hyperTonic solution for brain Edema) randomized trial

**DOI:** 10.1186/s40560-020-00449-0

**Published:** 2020-05-04

**Authors:** Ofer Sadan, Kai Singbartl, Jacqueline Kraft, Joao McONeil Plancher, Alexander C. M. Greven, Prem Kandiah, Cederic Pimentel, C. L. Hall, Alexander Papangelou, William H. Asbury, John J. Hanfelt, Owen Samuels

**Affiliations:** 1 Department of Neurology and Neurosurgery, Division of Neurocritical Care, Emory University Hospital and Emory University School of Medicine, 1364 Clifton Rd. NE, Atlanta, GA 30322 USA; 2 Department of Critical Care Medicine, Mayo Clinic, 5777 E Mayo Blvd, Phoenix, AZ 85054 USA; 3 School of Medicine, Emory University, 1364 Clifton Rd. NE, Atlanta, GA 30322 USA; 4 Department of Anesthesiology, Emory University Hospital and Emory University School of Medicine, 1364 Clifton Rd. NE, Atlanta, GA 30322 USA; 5 Department of Pharmacy, Emory University Hospital, 1364 Clifton Rd. NE, Atlanta, GA 30322 USA; 6 Department of Biostatistics and Bioinformatics, Emory University, 1364 Clifton Rd. NE, Atlanta, GA 30322 USA

**Keywords:** Cerebral edema, Subarachnoid hemorrhage, Hyperosmolar therapy, Hyperchloremia, Acute kidney injury, Neurocritical care

## Abstract

**Background:**

Recent reports have demonstrated that among patients with subarachnoid hemorrhage (SAH) treated with hypertonic NaCl, resultant hyperchloremia has been associated with the development of acute kidney injury (AKI). We report a trial comparing the effect of two hypertonic solutions with different chloride contents on the resultant serum chloride concentrations in SAH patients, with a primary outcome aimed at limiting chloride elevation.

**Methods:**

A low ChloridE hyperTonic solution for brain Edema (ACETatE) trial is a single-center, double-blinded, double-dummy, randomized pilot trial comparing bolus infusions of 23.4% NaCl and 16.4% NaCl/Na-acetate for the treatment of cerebral edema in patients with SAH. Randomization occurred when patients developed hyperchloremia (serum Cl^−^ ≥ 109 mmol/L) and required hyperosmolar treatment.

**Results:**

We enrolled 59 patients, of which 32 developed hyperchloremia and required hyperosmolar treatment. 15 patients were randomized to the 23.4% NaCl group, and 17 patients were randomized to the 16.4% NaCl/Na-acetate group. Although serum chloride levels increased similarly in both groups, the NaCl/Acetate group showed a significantly lower Cl^−^ load at the end of the study period (978mEq vs. 2,464mEq, *p* < 0.01). Secondary outcome analysis revealed a reduced rate of AKI in the Na-acetate group (53.3% in the NaCl group vs. 11.8% in the Na-acetate group, *p* = 0.01). Both solutions had similar effects on ICP reduction, but NaCl/Acetate treatment had a more prominent effect on immediate post-infusion Na^+^ concentrations (increase of 2.2 ± 2.8 vs. 1.4 ± 2.6, (*p* < 0.01)). Proximal tubule renal biomarkers differed in concentration between the two groups.

**Conclusions:**

Our pilot trial showed the feasibility and safety of replacing 23.4% NaCl infusions with 16.4% NaCl/Na-acetate infusions to treat cerebral edema in patients with SAH. The degree of hyperchloremia was similar in the two groups. 16.4% NaCl/Na-acetate infusions led to lower Cl^−^ load and AKI rates than 23.4% NaCl infusions. Further multi-center studies are needed to corroborate these results.

**Trial registration:**

clinicaltrials.gov # NCT03204955, registered on 6/28/2017

## Introduction

Aneurysmal subarachnoid hemorrhage (aSAH), a life-threatening condition that results from a rupture in a cerebral artery [1]. Although a relatively rare type of stroke, it carries a disproportionally high risk for mortality and morbidity. For example, even with treatment improvements in recent years, aSAH in-hospital mortality rates remain around 20% [2].

Aside from CNS-related complications such as hydrocephalus, cerebral edema, or delayed cerebral ischemia, systemic complications account for up to 40% of all deteriorations during inpatient care [3]. Acute kidney injury in aSAH patients is associated with a greater than four-fold increase in hospital mortality, along with less favorable neurological recovery in patients who survive [4–6].

Among critically ill patients, it has been suggested that hyperchloremia can precipitate or worsen AKI and increase the need for renal replacement therapy [7–9]. Prospective comparative studies between isotonic NaCl and balanced crystalloid solutions have consistently shown an association between isotonic NaCl infusions, an increased risk for AKI, and subsequent poor outcome in critically ill patients [10–13].

Hyperosmolar therapy is a cornerstone in the treatment of aSAH-related cerebral edema, which is necessary in approximately 20–30% of SAH patients [14]. Osmotherapy commonly includes infusions of hypertonic saline, which has a significantly higher chloride concentrations in comparison with standard NaCl 0.9% solution. It is of no surprise, therefore, that in a recently published retrospective analysis of SAH patients, we observed correlations between hyperchloremia and AKI, and between AKI and increased morbidity and mortality. We identified treatment with hypertonic NaCl as a risk factor for the development of hyperchloremia [5]. We were not, however, able to demonstrate a dose-response relationship between chloride load and the subsequent development of hyperchloremia. Instead, a great variability in chloride exposure was observed [5]. The large variability appeared to be related to a high incidence of cerebral salt-wasting syndrome in our study population, resulting in the need for extensive sodium and volume replacement with intravenous (IV) fluids, particularly with NaCl solutions of various concentrations [15].

Therefore, the question arises as to whether avoiding further worsening of hyperchloremia by differential use of hypertonic solutions might be a better strategy for the prevention of AKI in aSAH patients. At our institution, we began utilizing a compound solution that replaces part of the NaCl solution with sodium-acetate, offering a lower chloride load per dose. Further analysis of the previously published cohort allowed us to identify a Cl^−^ concentration of 109 mmol/L as a threshold that differentiates between patients at high versus low risk for AKI [16].

Focusing on at-risk patients, this trial aimed to reduce exacerbations of hyperchloremia in aSAH patients treated with hypertonic saline who developed mild hyperchloremia by using a double-blind, double-dummy, pragmatic approach.

## Methods

### Patient population and problem investigated

Our trial protocol has been published previously [16] and was approved by the Emory University institutional review board (IRB). We utilized a single-center double-blinded, double-dummy, pilot design to compare NaCl 23.4% and NaCl/Na-acetate 16.4% (Table 1) for the treatment of cerebral edema in patients with aneurysmal SAH and evolving hyperchloremia (serum Cl^−^ ≥ 109 mmol/L).

**Table 1:** Composition of the two hypertonic solutions compared in the trial

	NaCl (standard treatment) group	NaCl/Na-acetate (alternate treatment) group
Solution components (per dose)	Sodium chloride	Sodium chloride and sodium acetate
Na Concentration [%]	23.4	16.4
NaCl pre-mixed solution, 4 mEq/ml [ml]	30	20
Na**-**acetate pre-mixed solution, 2 mEq/ml [ml]	0	30
Volume [ml]	30	50
Sodium content [mEq/dose]	120	140
Chloride content [mEq/dose]	120	80
Acetate content [mEq/dose]	0	60

All patients admitted to the Emory University Hospital Neuroscience Critical Care Unit, Atlanta, GA, between June 25, 2017, and June 24, 2018, were screened. Adult patients with a diagnosis of aSAH, or their legally authorized representatives, were approached for consent, excluding those with a poor prognosis (e.g. brain death, or progressing towards brain death) and known end-stage renal disease.

### Intervention and comparison

After enrollment, patients who required hyperosmolar therapy and had developed hyperchloremia (i.e., Cl^−^ ≥ 109 mmol/L) were ultimately randomized to infusions of 23.4% NaCl or 16.4% NaCl/Na-acetate. As a pragmatic protocol, the decision on initiation of hyperosmolar therapy was made by the attending physician. The indications were either cerebral edema evident on imaging, or increase in intracranial pressure, or both. All other IV fluids administered to the patients were balanced IV solution (PlasmaLyte®), in all groups, apart from carrier solution needed for IV medications. All IV treatments were used to calculate the sodium and chloride exposure. For randomization to NaCl or the NaCl/Na-acetate group (1:1 ratio), we used random block sizes stratified by (a) serum creatinine level on admission (normal vs. abnormal according to our clinical laboratory normal range) and (b) SAH Hunt & Hess score, i.e., low [1, 2] vs. high [3–5]. Due to volumetric variations between the treatment arms, we used a double-dummy approach. Two IV bags were administered at the same time via different ports: one with the hypertonic solution and the other with PlasmaLyte® matching the volume of the solution from the other trial group. Central and systemic complications rates were followed, by using commonly accepted definitions (vasospasm/DCI [17], ARDS [18], and sepsis [19]). Patients’ functional outcomes were assessed with the modified Rankin scale at discharge, and again at 90 days, either during clinic visits or by telephone interview.

We intended to randomize 60 patients for this pilot trial (30 patients per group), to have a 63% power to detect a six-point difference in the change in serum Cl^−^ levels between treatment groups (two-sided test, type I error rate 0.05), based on our retrospective data. Our data analysis followed the intent-to-treat principle.

Urine samples were collected and frozen in a – 70 ^o^C freezer. We analyzed urine samples for biomarkers of renal injury (IL-18, KIM-1, NGAL, and cystatin C), using a multiplexed immunoassay (Meso-Scale Discovery, Gaithersburg, MD). Data were analyzed by laboratory personnel blinded to trial conditions using MSD integrated data analysis software that converts ECL signal to pg/mL values based on standard curves of calibrator proteins.

### Outcome

The primary outcome was to assess longitudinal serum chloride concentration changes in aSAH patients treated with either NaCl 23.4% (30 mL) or NaCl/Na-acetate 16.4% (50 mL), between the pre-randomization concentration and the highest result following it during the admission. Secondary outcomes included new-onset AKI during hospital admission as defined by an increase in serum creatinine and/or a decrease in urine output according to the KDIGO criteria [20], incidence of all causes of in-hospital mortality, also counting withdrawal of treatment or discharge to a hospice facility, and assessment of the treatment effect of NaCl/Na-acetate 16.4% and NaCl 23.4% for intracerebral hypertension.

### Statistical analysis

Continuous data are presented as mean ± SD or as median ± Q1–Q3. Categorical data are presented as counts and percentages. Continuous outcomes were compared using two-sample *t* tests or ANOVA with post hoc Scheffe testing where appropriate. The Pearson chi-square test was used to compare binary proportions. Risk factors associated with AKI were assessed with univariate logistic regression analysis. Results are given as odds ratios (OR) with 95% confidence intervals. Statistical analyses were done using SPSS v. 25 (IBM, NY). *p* < 0.05 was considered significant.

## Results

### The patient cohort

**Fig. 1:**
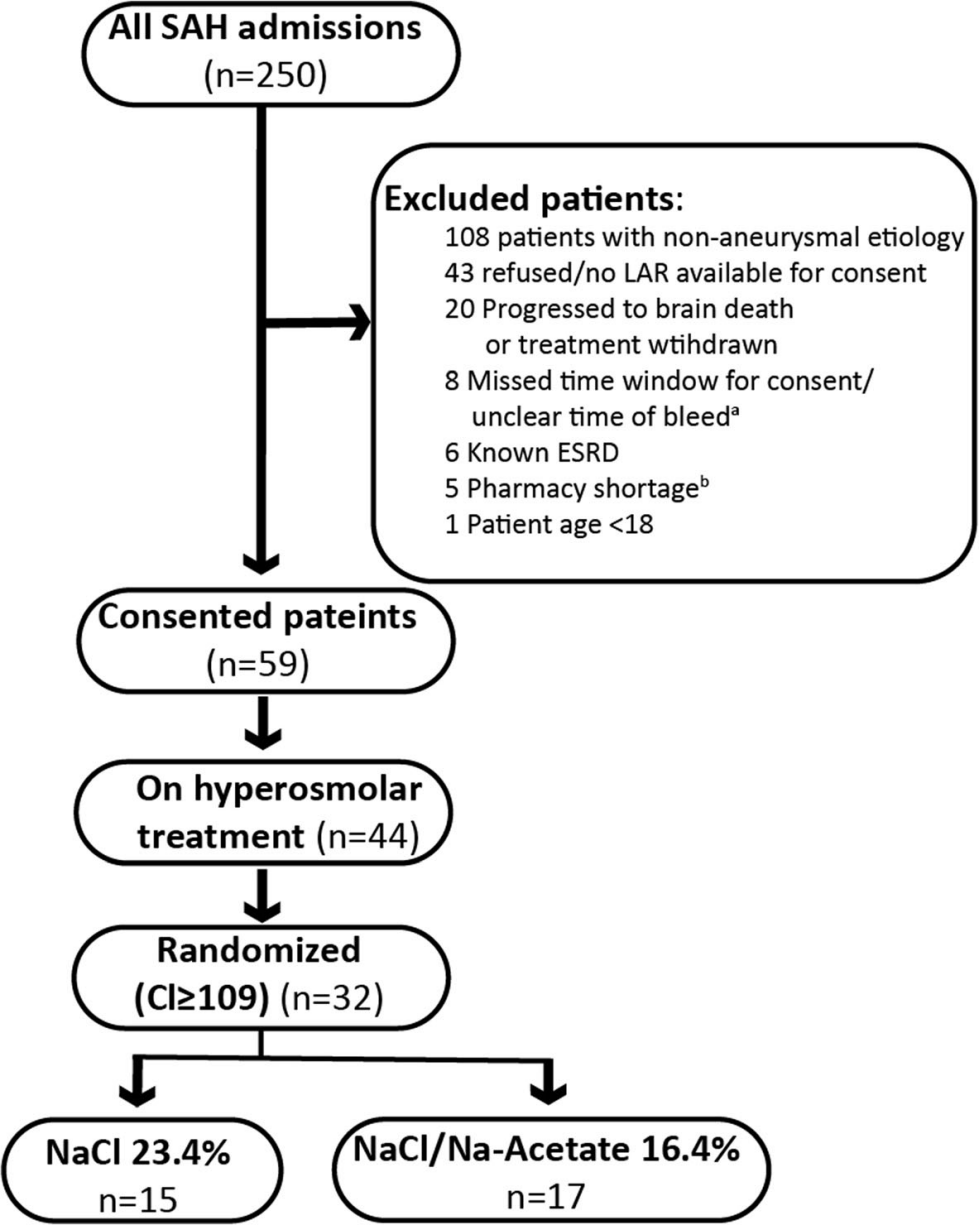
Patient allocation scheme. ^a^As per the protocol, consent was obtained within 48 h of admission, when symptoms began within a day of admission. In cases where the symptom onset was unclear, patients were excluded. ^b^During the study there was national shortage of Na-acetate solution, which led to a temporary delay in recruitment. LAR, legally authorized representative

Within 1 year, 250 patients with a diagnosis of SAH were admitted and screened. Of those, 59 patients were consented for the trial (Fig. 1). Patients’ demographic information, relevant risk factors, severity of illness, and surgical treatment are detailed in Table 2. There was no statistically significant difference between the two randomized groups in all of the captured parameters. Angiography was performed on admission to all patients, including those who required clip ligation for the securing the aneurysm. Recruitment was stopped at 1 year due to lack of funding and therefore was short of the enrollment target.

**Table 2:** Demographic information of the study cohort. There were no statistically significant differences between the two randomized groups in terms of the various clinical parameters. Results are presented as percentage with 95% confidence interval. *H&H* Hunt and Hess, *SOFA* sequential organ failure assessment score, *HTN* hypertension, *DM* diabetes mellitus, *CKD* chronic kidney disease

Parameter	All (*n* = 59)	Non-randomized (*n* = 27)	NaCl (*n* = 15)	NaCl/Na-acetate (*n* = 17)
Age	53.4 + 13.4	53.2 + 14.1	56.3 + 13.4	51.4 + 13.7
Gender (% female)	76.3% [64.3–85.7]	59.2% [40.6–76.1]	80% [55.6–94]	100%
H&H 1–2	44.1% [31.2–57.6]	66.7% [46.0–83.5]	20.0% [6.0–44.4]	29.4% [12.2–53.0]
H&H 3	39.0% [26.5–52.6]	29.6% [15.1–48.2]	66.7% [41.6–86.0]	29.4% [12.2–53.0]
H&H 4	15.3% [7.8–26.0]	3.7% [0.4–16.0]	13.3% [2.9–36.3]	35.3% [16.3–58.9]
H&H 5	1.7% [0.2–7.6]		0%	5.9% [0.6–24.4]
GCS on admit (median + IQR)	14 [11–15]	15 [13.5–15]	14 [7–15]	13 [7–15]
SOFA score on admit (median ± IQR)	1 [0–3]	1 [0–2]	2 [1–4.5]	2 [1–3]
HTN	47.5% [34.3–60.9]	40.7% [23.9–59.4]	53.3% [29.4–76.1]	52.9% [30.3–74.6]
CAD	6.8% [1.9–16.5]	3.7% [0.4–16.0]	13.3% [2.9–36.3]	5.9% [0.6–24.4]
DM	11.9% [4.9–22.9]	11.1% [3.2–26.8]	13.3% [2.9–36.3]	11.8% [2.5–32.7]
Smoker	47.5% [34.3–60.9]	55.6% [37.1–72.9]	40.0% [18.8–64.7]	41.2% [20.7–64.4]
CKD	1.7% [0.0–9.1]	0%	6.7% [0.7–27.2]	0%
EtOH abuse	10.2% [3.8–20.8]	14.8% [5.2–31.5]	6.7% [0.7–27.2]	5.9% [0.6–24.4]
Illicit drug use	25.4% [15.0–38.4]	29.6% [15.1–48.2]	6.7% [0.7–27.2]	35.3% [16.3–58.9]
First creatinine (mean ± SD)	0.80 + 0.31	0.82 + 0.27	0.79 + 0.26	0.76 + 0.42
Surgical approach to the aneurysm (% treated with endovascular approach)	69.5% [57..0–80.1]	81.5% [64.1–92.6]	66.7% [41.6–86.0]	52.9% [30.3–74.6]

Hypertonic saline was used in 74.6% of all patients. Of the 44 patients who received hypertonic saline, 33 developed hyperchloremia. There was one protocol deviation: one patient meeting randomization criteria was not randomized to a treatment arm due to a clerical error. Therefore, a total of 32 patients were randomized, 15 to the NaCl group and 17 to the NaCl/Na-acetate group. Pre-randomization, the baseline serum electrolytes were similar in both groups: Na^+^ was 142.8 ± 2.3 mmol/L in the NaCl group compared with 142.2 ± 3.1 mmol/L in the Na-acetate one; Cl^−^ concentrations were 111.8 ± 2.8 mmol/L compared with 112.1 ± 2.6 mmol/L, respectively; and HCO_3_^−^ concentrations were 23.4 ± 3.1 mmol/L compared with 22.4 ± 3.2 mmol/L, respectively. Randomization occurred on average 3.1 ± 0.8 days post admission in the NaCl group, and 2.8 ± 1.1 in the Na-acetate group (*p* = 0.5).

### The effect of hypertonic solution on serum electrolytes

**Fig. 2:**
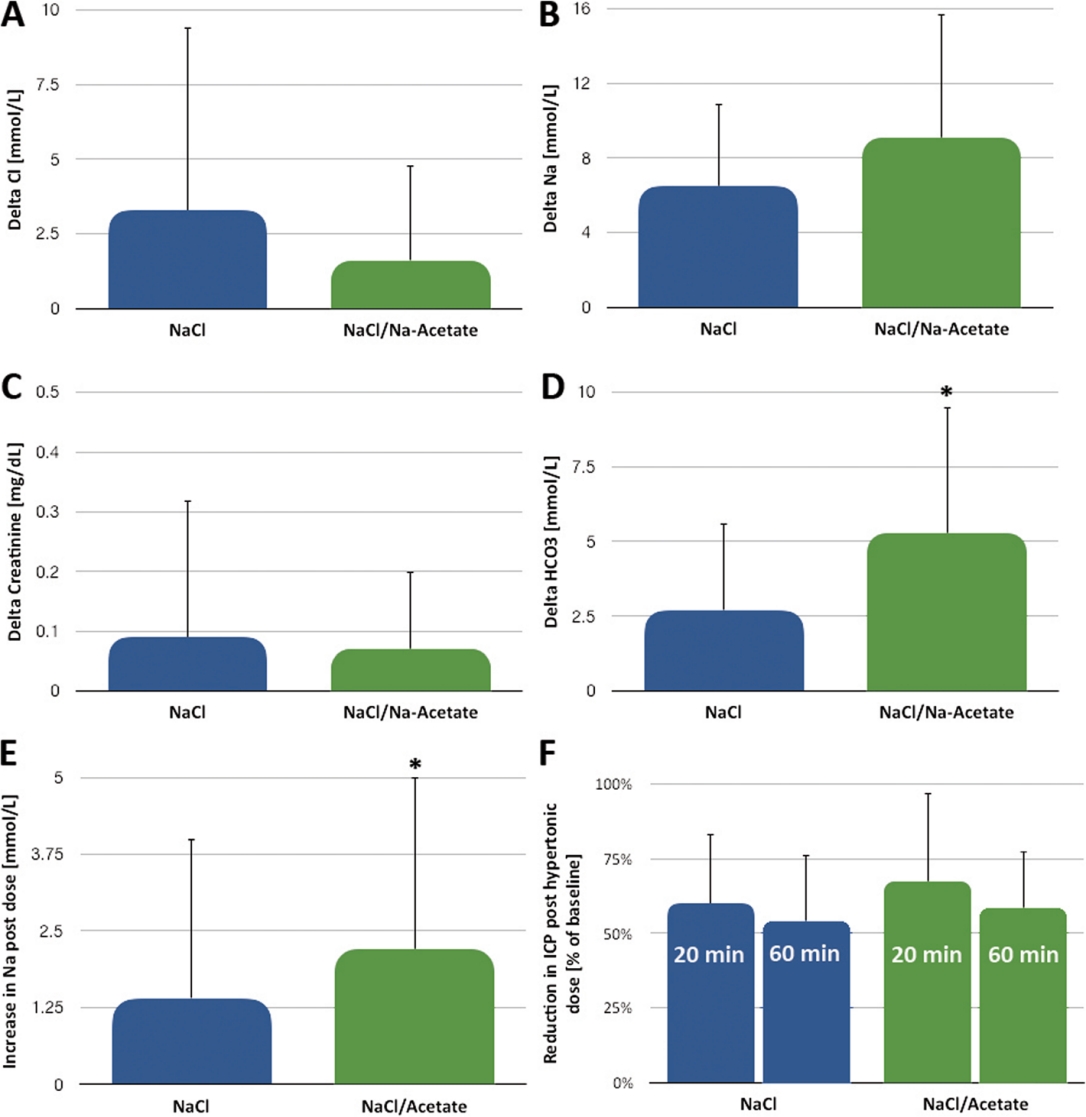
The effect of hypertonic solution content on serum electrolytes. **a** ∆ Chloride between time of randomization and the highest recorded value post randomization during the ICU stay was marginally lower in the NaCl/Na-acetate group, yet not statistically significant. **b** ∆ Sodium between time of randomization and the highest value post randomization during ICU stay was marginally higher in the NaCl/Na-acetate group, yet not in a statistically significant manner. **c** ∆ creatinine was similar between the groups. **d** ∆ Bicarbonate (HCO_3_) was higher in the NaCl/Na-acetate group in a statistically significant manner. **e** Change in sodium pre- and post-dose was higher in the NaCl/Na-acetate group. **f** Reduction in ICP was similar between the groups at 20 and 60 min post-administration. *p < 0.05

There was a non-significant trend (*p* = 0.36, Fig. 2a) towards a smaller increase in serum Cl^−^ in the Na-acetate group (1.6 ± 3.2 mmol/L) when compared with the NaCl group (3.3 ± 6.1 mmol/L). The increase in serum Na^+^ was higher in the Na-acetate group, although not statistically significant (6.5 ± 4.4 vs. 9.1 ± 6.6 mmol/L, *p* = 0.18, Fig. 2b). Patients randomized to the Na-acetate group also had mildly higher mean serum Na^+^ concentrations throughout their ICU stay than patients in the NaCl group (on average: 142.3 + 3.4 mmol/L vs. 140.1 + 3.6, *p* = 0.09). Mean serum Cl^−^ concentrations (across all of the ICU stay) were nearly identical (Na-acetate group 106.0 + 3.4, NaCl group 106.2 + 2.5 mmol/L, *p* = 0.8). Mean serum HCO_3_^−^ concentrations were higher in the Na-acetate group (26.3 ± 1.8 mmol/L) than in the NaCl group (25.0 ± 1.8 mmol/L, *p* = 0.05). The increase in serum HCO_3_^−^ was also significantly higher (*p* < 0.05, Fig. 2d) in the Na-acetate group (5.3 ± 4.2 mmol/L) than in the NaCl group (2.7 ± 2.9 mmol/L).

### The immediate effects of the hypertonic solutions

When treated with hypertonic solutions, patients’ sodium is measured every 4–6 h. One dose of 23.4% NaCl increased serum Na^+^ levels by 1.4 ± 2.57 mmol/L within 4 h, while one dose of 16.4% NaCl/Na-acetate raised serum Na^+^ concentrations by 2.18 ± 2.81 mmol/L (*p* < 0.01, Fig. 2e).

In cases of intracranial hypertension, i.e., a sustained ICP increase of more than 20 mmHg, infusions of 23.4% NaCl decreased ICP to 59.9 ± 23.1% of baseline within 20 min. Infusions of 16.4% NaCl/Na-acetate lowered ICP to 67.4 ± 22.5% of baseline (18 events, *p* = 0.6, Fig. 2f). After 1 h, the reduction in ICP was sustained: 58.6 ± 18.7% of baseline in the NaCl group and at 53.9 ± 29.6% in the NaCl/Na-acetate group (*p* = 0.4).

### The effect of hypertonic solution on renal function

**Fig. 3:**
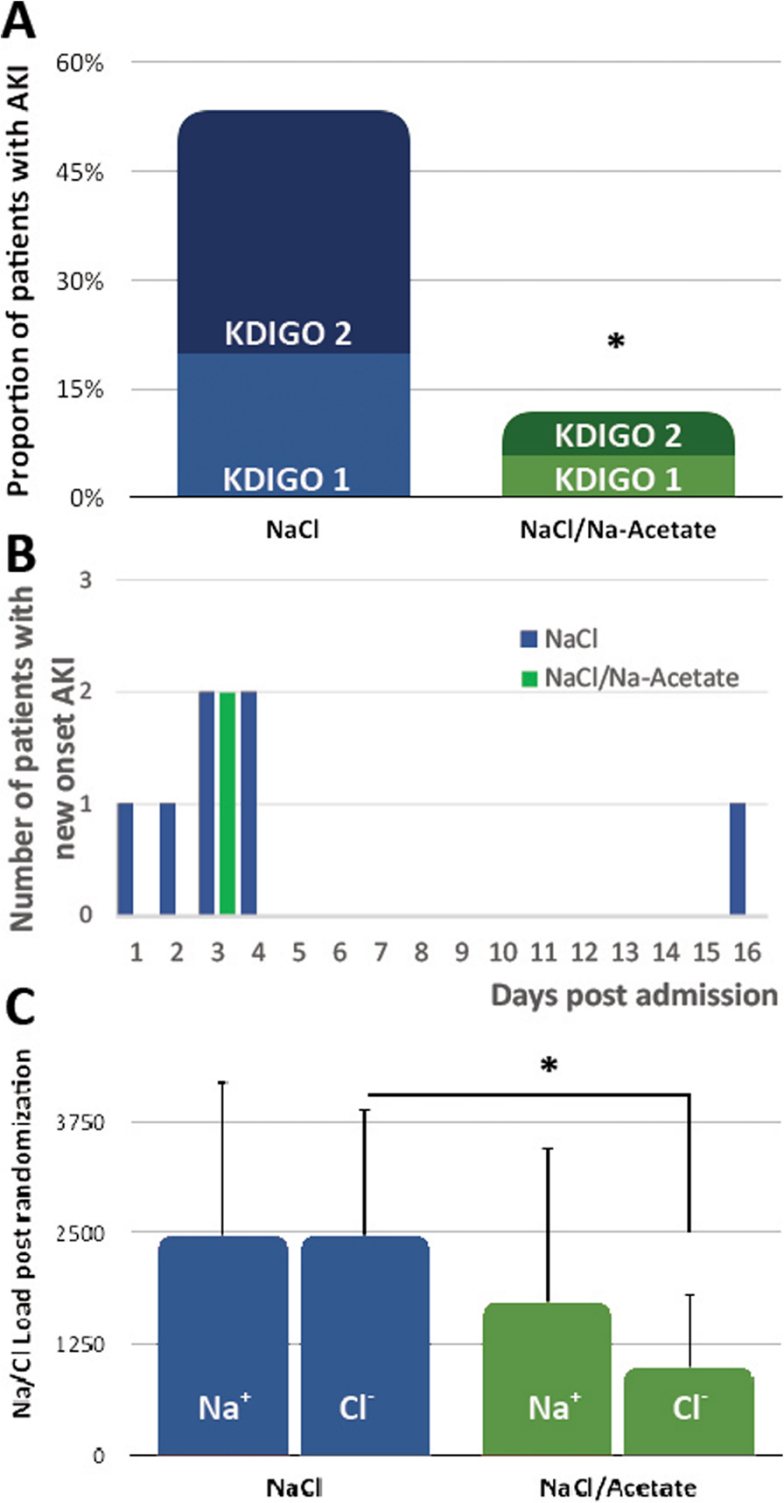
The effect of hypertonic solution on renal function and ICP reduction. **a** The rate of AKI was lower in the NaCl/Na-acetate group as compared with the NaCl group in an intention to treat analysis. **b** Comparison of Na^+^/Cl^−^ loads with the study intervention doses, post-randomization. **c** Histogram of AKI frequency by group of treatment and hospitalization day. *p < 0.05. AKI, acute kidney injury; KDIGO, Kidney Disease: Improving Global Outcomes grading for AKI

AKI occurred in 23.7% of this cohort (see KDIGO grading in Fig. 3a). AKI events occurred on median hospital day 3 (IQR2–7, Fig. 3b). Hyperchloremia preceded AKI in most cases (eight of the fourteen cases of AKI). The rate of AKI was higher in the NaCl group as compared with the NaCl/Na-acetate group (53.3% vs. 11.8% respectively, *p* = 0.01, Fig. 3a). In the group that was not randomized, AKI occurred in 14.8% of the cases, all KDIGO grade 1. Time between the appearance of hyperchloremia and the appearance of AKI was very short with a median of 0.75 [0.13–2.75] days between the two events—a result observed in both randomized groups. Hence, AKI post randomization occurred in 13.3% of the NaCl group and in 0% in the NaCl/Na-acetate one. Hyperchloremia as a biomarker for elevated risk of AKI achieved a sensitivity of 36.4% and specificity of 16.2%. In the NaCl group, 37.5% of AKI events were grade 1, and 62.5% were grade 2. In the Na-acetate group, one event was grade 1 and the other grade 2.

Post randomization, the Na-acetate group received a lower number of doses in total (12.2 ± 10.3 on average), compared with the NaCl group (20.5 ± 14.4, *p* = 0.07). However, when normalized, both groups received a similar number of hypertonic solution doses per day of treatment: 2.2 ± 0.8 doses per day in the NaCl group and 1.8 ± 0.9 in the NaCl/Na-acetate one (*p* = 0.12). The total sodium load from hypertonic solutions post randomization was also similar between the groups, while the chloride load was more than doubled in the NaCl group compared with the NaCl/Na-acetate solution (*p* < 0.01, Fig. 3c).

When correlating different risk factors to AKI, using a binary regression analysis, the only parameters that were correlated in a statistically significant manner were treatment with NaCl/Na-acetate (which demonstrated a protective effect) and ∆ chloride (Table 3).

**Table 3:** Binary logistic regression correlating between clinical parameters and AKI (*p < 0.05)

Parameter	OR [CI 95%]
Age	0.97 [0.92–1.03]
Gender	5.25 [0.42–66.22]
Hypertension	0.83 [0.19–3.72]
Diabetes mellitus	0.70 [0.06–7.74]
Coronary artery disease	1.11 [0.09–13.89]
Aneurysm treatment approach	0.51 [0.11–2.53]
Treatment with NaCl/Na-acetate*	0.18 [0.02–0.70]
∆ Chloride*	1.32 [1.04–1.67]
∆ Sodium	1.03 [0.91–1.17]
Chloride load post randomization	1.00 [1.00–1.00]
Sodium load post randomization	1.00 [1.00–1.00]

With the exception of AKI, there was no difference in major ICU-related complications between the randomized groups (Additional file 1: Table S1). Although there was no difference between the randomized groups, there was a clear difference between the enrolled patients that were randomized versus those who were not. The latter had lower rates of cerebral vasospasm, DCI, and all of the systemic complications monitored.

### The effect of hypertonic solution on patient outcomes

In terms of patient outcome, comparison between the two randomized groups (NaCl vs. NaCl/Na-acetate) showed no difference in outcome (survival and functional outcome) in this small cohort (Additional file 2: Table S2).

### Change in urine AKI biomarkers

**Fig. 4:**
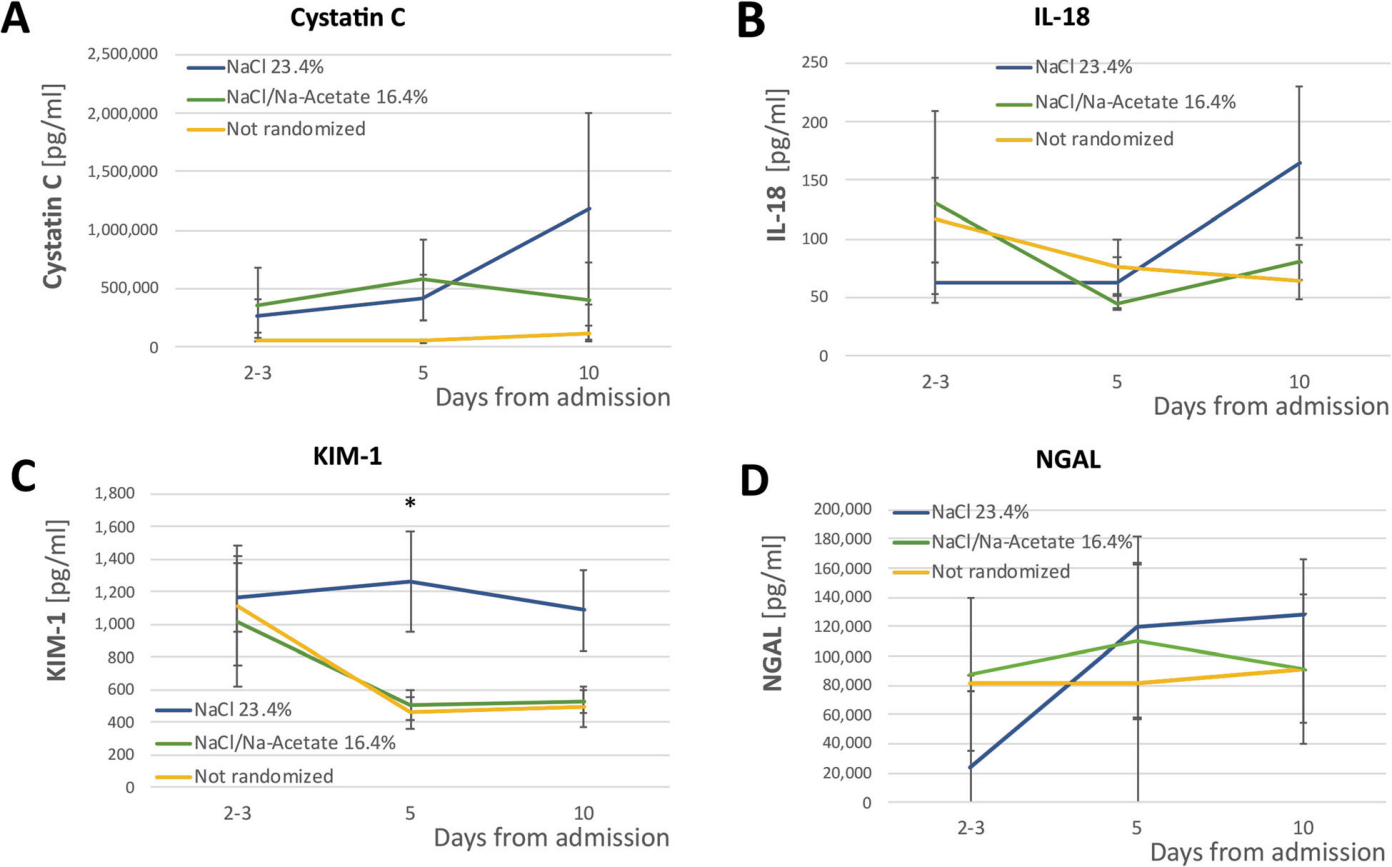
Change in urine AKI biomarkers along the first 10 days of admission. **a**–**d** Change in biomarker concentration between the different treatment groups (non-randomized, randomized to receive NaCl 23.4% and randomized to receive NaCl/Na-acetate 16.4%). The first time point is days 2–3, which was the day of consent, which was either admission day 2 or 3. *p < 0.05 between the groups. AKI, acute kidney injury

We further measured four urine biomarkers of AKI to study the development of AKI. Samples were collected on the day of recruitment (days 2–3 of admission), day 5, and day 10. Differences in KIM-1 concentrations were only apparent at day 5. NGAL, cystatin C, and IL-18 did not demonstrate any significant differences between the groups (Fig. 4).

## Discussion

Our pilot study demonstrated the feasibility, safety, and effectiveness of replacing infusions of 23.4% NaCl with infusions of 16.4% NaCl/Na-acetate to treat cerebral edema in patients with aSAH. When compared to NaCl, NaCl/Na-acetate infusions yielded a non-significant trend towards lower ∆ serum Cl^−^ concentrations. Our trial was budgeted to run for only 1 year. It had to be stopped early for financial reasons and was intermittently interrupted by an unexpected national shortage of sodium-acetate solution. This prevented us from enrolling all of the intended 60 patients within 1 year. Consequently, the trial ended up underpowered for the primary outcome, a reduction in ∆ serum chloride.

Hyperosmolar therapy is a mainstay in the treatment of acute brain injuries leading to cerebral edema. For the last few decades, hypertonic saline and mannitol have been the two main hyperosmolar agents used in clinical practice. Available studies suggest a preference for the use of hypertonic saline [21, 22] because of its favorable efficacy, though evidence is limited. There remain concerns about the morbidity associated with hypertonic saline solutions, in particular hyperchloremic acidosis and AKI [5].

Stimulated by the development of balanced solutions that are increasingly replacing NaCl 0.9% and demonstrating reduced AKI rates [12, 13], we set out to mix two existing hypertonic solutions, namely NaCl and Na-acetate. The solution created was used off-label in our unit for the treatment of severe hyperchloremia in the setting of brain edema necessitating hyperosmolar treatment. The components are “off-the-shelf” solutions that were mixed by the hospital pharmacy for off-label use. The result was a solution that had a slightly lower sodium concentration (23.4 vs 16.4%) and a slightly higher volume (50 ml compared with 30 ml), yet delivered a higher amount of sodium per dose, with a smaller amount of chloride. Acetate, the alternative electrolyte used, serves as an energy source, mainly for hepatocytes, in a normal physiological state [23]. Acetate is widely employed in various clinical settings, in particular as a component of parenteral nutrition. Germane to the patients with acute brain injury, sodium-acetate solutions have been proven to be beneficial for the treatment of cerebral edema [24]. Another option for hypertonic solution without chloride that was recently studied is sodium-lactate. The latter was evaluated in several small-scale clinical trials in the setting of acute brain injury [25, 26] with favorable results. It is currently unknown what is the best counterpart for sodium in terms of efficacy and safety.

Our study design aimed to identify and then randomize patients with a high risk for AKI, i.e., patients with hyperchloremia. Our prior results suggested that patients with chloride of 109 mmol/L or above are those with the highest risk for AKI, and therefore, this threshold was selected [16]. Serum Cl^−^ was used as a biomarker for AKI risk and triggered our randomization. The selection for randomization based on the need for hyperosmolar therapy and mild hyperchloremia led to a selection of patients with higher rates of systemic and central complications (e.g., DCI, days of ventilation, etc.), regardless of the treatment arm to which these patients were randomized. Similarly, the outcomes of the randomized patients were worse in any parameter compared to those who were not randomized, probably due to their poorer baseline status, such as higher admission H&H scale.

Serum Cl^−^ was elevated prior to most AKI events, although the time gap was rather small. Therefore, only some AKI patients received the study intervention before AKI could be diagnosed by standard clinical criteria. Chloride failed in its role in two different aspects: first, its sensitivity and specificity for prediction of the risk for AKI were low; and second, the hyperchloremic event happened too close from a temporal standpoint to the AKI event. Therefore, only in some AKI cases did the patient receive the study intervention before AKI occurred. Interestingly, there were no AKI events post-randomization in the NaCl/Na-acetate group, but 25% of AKI events in the NaCl group were diagnosed post-randomization. It might be that the hypertonic content impacts delayed AKI, and not early events. Overall, the results suggest that a future trial should randomize patients early, and not wait for hyperchloremia to develop.

The primary outcome of this study was the reduction in ∆ serum chloride post randomization. While underpowered for this purpose, the results did show a non-significant trend toward a lower ∆ in the NaCl/Na-acetate group. However, the data also demonstrated a trend toward an increase in ∆ sodium in the NaCl/Na-acetate group, and an even more pronounced difference when measuring the ∆ between the pre- and post-dose sodium serum concentrations. These results suggest that the absolute content of sodium per dose is more significant than the change in percentage from 23.4 to 16.4%, in our particular case. The results also show that when administered specifically for elevated ICP, the performance of both solutions is comparable. In the absence of adverse effects (similar efficacy, similar long-term outcome, reduced rate of AKI), the results may suggest that future trials could test an alternative hypertonic solution without further selecting higher risk patients, as this study did.

From a mechanistic standpoint, the study evaluated a panel of urine biomarkers and their association with AKI. The panel allowed for a better understanding of the pathophysiology underlying AKI in the settings of SAH and hyperchloremia. Measures of IL-18, an inflammatory biomarker [27]; cystatin-C, a marker of glomerular filtration rate; and NGAL, a distal nephron injury marker [28], did not differ between the groups. However, a signal was found in the measurement of KIM-1, a marker of proximal tubular injury [28]. Interestingly, patients who were randomized to the NaCl 23.4% group showed a marked increase in urinary NGAL up to day 5—a change that was not apparent in the NaCl/Na-acetate group, even though per time point, there was no statistically significant difference between the groups. In the case of KIM-1, the best differentiation between the groups was possible only post facto, since the median appearance of AKI in this cohort was on day 3.

Our study was severely limited by the number of patients enrolled. The study was funded for 1 year and aimed to randomize 60 patients. We were, however, only able to randomize 32. The lack of power does allow us to formulate hypotheses but not to generalize our results.

To this end, our pilot double-blinded, double-dummy, single-center clinical suggests that 16.4% NaCl/Na-acetate is a safe and effective alternative to 23.4% NaCl to induce hypernatremia and reduce ICP in SAH patients with cerebral edema. Our study further showed that NaCl/Na-acetate may modify the risk of AKI by reducing chloride load. All of these require further scrutiny in appropriately powered, randomized, multi-center trials.

## Conclusion

This pilot trial showed the feasibility and safety of replacing 23.4% NaCl infusions with a chloride-lean, 16.4% NaCl/Na-acetate infusions to treat cerebral edema in patients with SAH. Although the chloride load in patients receiving the alterative solution was lower, the degree of hyperchloremia was similar in the two groups. 16.4% NaCl/Na-acetate infusions was associated with lower AKI rates than 23.4% NaCl infusions, and had a similar effect on ICP reduction. Further multi-center studies are needed to corroborate these results.

## Supplementary information

**Additional file 1: Table S1.** ICU complications rate. No statistically significant difference was noted between the two treatment groups.

**Additional file 2: Table S2.** Patient outcomes. No statistically significant difference was noted between the two treatment groups. Ten patients were lost to long-term (90 day) follow up. mRS – Modified Rankin Scale.

## Data Availability

The datasets used and/or analyzed during the current study are available from the corresponding author on reasonable request.

## Abbreviations

AKI: Acute kidney injury
ARDS: Acute respiratory distress syndrome
aSAH: Aneurysmal subarachnoid hemorrhage
CNS: Central nervous system
DCI: Delayed cerebral ischemia
ICP: Intracranial pressure
IRB: Institutional review board
KDIGO: Kidney Disease: Improving Global Outcomes
LAR: Legally authorized representative
NGAL: Neutrophil gelatinase-associated lipocalin
