# Correction to: Low-chloride- versus high-chloride-containing hypertonic solution for the treatment of subarachnoid hemorrhage–related complications: The ACETatE (A low ChloriE hyperTonic solution for brain Edema) randomized trial

**DOI:** 10.1186/s40560-020-00485-w

**Published:** 2020-09-01

**Authors:** Ofer Sadan, Kai Singbartl, Jacqueline Kraft, Joao McONeil Plancher, Alexander C. M. Greven, Prem Kandiah, Cederic Pimentel, C. L. Hall, Alexander Papangelou, William H. Asbury, John J. Hanfelt, Owen Samuels

**Affiliations:** 1 Department of Neurology and Neurosurgery, Division of Neurocritical Care, Emory University Hospital and Emory University School of Medicine, 1364 Clifton Rd. NE, Atlanta, GA 30322 USA; 2 Department of Critical Care Medicine, Mayo Clinic, 5777 E Mayo Blvd, Phoenix, AZ 85054 USA; 3 School of Medicine, Emory University, 1364 Clifton Rd. NE, Atlanta, GA 30322 USA; 4 Department of Anesthesiology, Emory University Hospital and Emory University School of Medicine, 1364 Clifton Rd. NE, Atlanta, GA 30322 USA; 5 Department of Pharmacy, Emory University Hospital, 1364 Clifton Rd. NE, Atlanta, GA 30322 USA; 6 Department of Biostatistics and Bioinformatics, Emory University, 1364 Clifton Rd. NE, Atlanta, GA 30322 USA

**Correction to: J Intensive Care 8, 32 (2020)**

**https://doi.org/10.1186/s40560-020-00449-0**

Following the publication of the original article [1], it was noted that Fig. 3b had an erroneous graph. The correct Fig. 3 has been included in this correction. The authors apologize for this error.

**Fig. 3:**
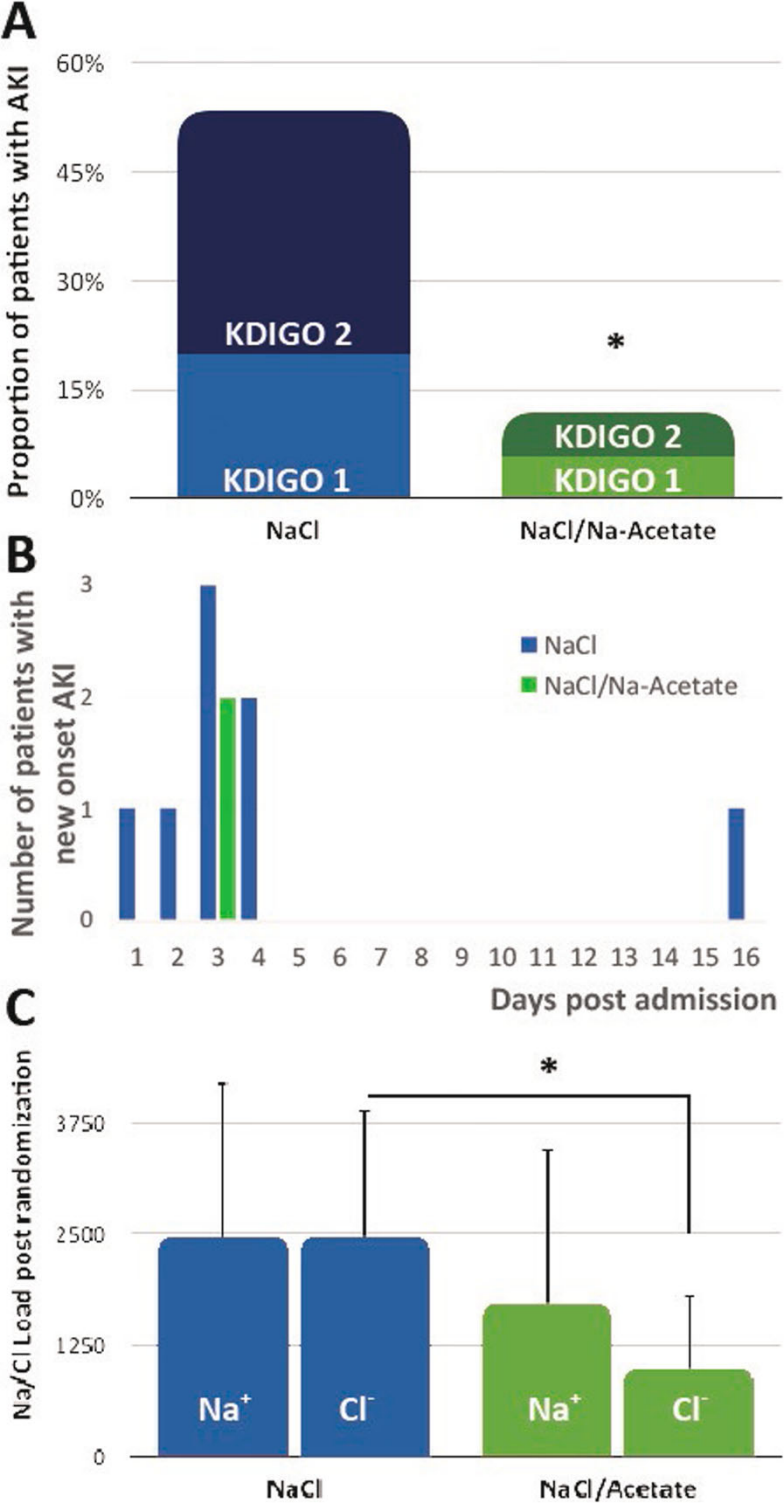
The effect of hypertonic solution on renal function and ICP reduction. **a** The rate of AKI was lower in the NaCl/Na-acetate group as compared with the NaCl group in an intention to treat analysis. **b** Comparison of Na^+^/Cl^−^ loads with the study intervention doses, post-randomization. **c** Histogram of AKI frequency by group of treatment and hospitalization day. *p < 0.05. AKI, acute kidney injury; KDIGO, Kidney Disease: Improving Global Outcomes grading for AKI
