# Supplementary material for: Low-chloride- versus high-chloride-containing hypertonic solution for the treatment of subarachnoid hemorrhage–related complications: The ACETatE (A low ChloriE hyperTonic solution for brain Edema) randomized trial
Source: J Intensive Care. 2020 May 4;8:32. doi: 10.1186/s40560-020-00449-0 (PMC7197130; doi:10.1186/s40560-020-00449-0)
Supplement: Supplementary file 1 — Additional file 1: Table S1. ICU complications rate. No statistically significant difference was noted between the two treatment groups. [file 40560_2020_449_MOESM1_ESM.docx]

| Parameter | All | Non-randomized | NaCl | NaCl/Na-Acetate |
| --- | --- | --- | --- | --- |
| Vasospasm | 52.5% [40.7-65.9] | 19.2% [7.7-37.1] | 80.0% [55.6-94.0] | 82.4% [60.0-94.8] |
| Radiological DCI | 23.7% [14.3-35.7] | 7.4% [1.6-21.7] | 53.3% [29.4-76.1] | 23.5% [8.5-46.7] |
| Ventilated (days on vent) | 52.5% [39.9-64.9]  (7.0+12.5) | 22.2% [9.8-40.2]  (0.3+0.7) | 80.0% [55.6-90.0]  (12.2+15.1) | 76.5% [53.3-91.5]  (12.9+15.2) |
| ARDS | 22.0% [13.0-33.8] | 0% | 46.7% [23.9-70.6] | 35.3% [16.3-58.9] |
| Sepsis | 13.6% [6.6-24.0] | 0% | 13.3% [2.9-36.3] | 35.3% [16.3-58.9] |
| DVT | 5.1% [1.5-13.0] | 3.7% [0.4-16.0] | 6.7% [0.7-27.2] | 5.9% [0.6-24.4] |

Table 1S: ICU complications rate. No statistically significant difference was noted between the two treatment groups.
