# Supplementary material for: Low-chloride- versus high-chloride-containing hypertonic solution for the treatment of subarachnoid hemorrhage–related complications: The ACETatE (A low ChloriE hyperTonic solution for brain Edema) randomized trial
Source: J Intensive Care. 2020 May 4;8:32. doi: 10.1186/s40560-020-00449-0 (PMC7197130; doi:10.1186/s40560-020-00449-0)
Supplement: Supplementary file 2 — Additional file 2: Table S2. Patient outcomes. No statistically significant difference was noted between the two treatment groups. Ten patients were lost to long-term (90 day) follow up. mRS – Modified Rankin Scale. [file 40560_2020_449_MOESM2_ESM.docx]

| Parameter | All | Non-randomized | NaCl | NaCl/Na-Acetate |
| --- | --- | --- | --- | --- |
| ICU days | 18.4+9.3 | 12.6+4.4 | 23.3+10.1 | 23.2+9.4 |
| mRS ≤2 at d/c | 32.2% [21.4-44.8] | 51.9% [33.6-69.7] | 13.3% [2.9-36.3] | 17.6% [5.2-40.0] |
| 90d survival | 97.9% [90.7-99.8] | 100% | 91.7% [67.2-99.1] | 100% |
| 90d ≤2 mRS | 53.1% [39.2-66.5] (10 missing) | 81.8% [62.4-93.5]  (5 missing) | 30.8% [11.4-57.7]  (2 missing) | 28.6% [10.5-28.6]  (3 missing) |

Table 2S: Patient outcomes. No statistically significant difference was noted between the two treatment groups. Ten patients were lost to long-term (90 day) follow up. mRS – Modified Rankin Scale.
